# Enhancement of Clathrate Hydrate Formation Kinetics Using Carbon-Based Material Promotion

**DOI:** 10.3389/fchem.2020.00464

**Published:** 2020-06-16

**Authors:** Yuan-Mei Song, Ru-Quan Liang, Fei Wang, Jian-Hui Shi, Deng-Bo Zhang, Liu Yang

**Affiliations:** ^1^The School of Mechanical & Vehicle Engineering, Linyi University, Linyi, China; ^2^Shandong Engineering Laboratory for Preparation and Application of High-Performance Carbon-Materials, College of Electromechanical Engineering, Qingdao University of Science & Technology, Qingdao, China

**Keywords:** gas hydrates, methane storage, efficient promoter, carbon-based materials, kinetic promotion

## Abstract

Although hydrate-based technology has been considered as a safe and environmentally friendly approach for gas storage and transportation in recent decades, there are still inherent problems during hydrate production, such as a long induction time, slow formation kinetics, and limited hydrate storage capacity. Attempts to resolve these issues have resulted in the development of various kinetics promoters, among which carbon-based materials have become one of the most attractive owing to their unique promotion effect. Herein, results on promotion by bulk wetted carbon materials in the forms of a packed bed, carbon particles in a suspension, and nano-carbon materials in a nanofluid are collected from the published literature. Meanwhile, the promotion mechanisms and influencing factors of the carbon-based promoters are discussed. The purpose of this mini-review is to summarize recent advances and highlight the prospects and future challenges for the use of carbon-based materials in hydrate production.

## Introduction

Natural gas hydrate, also referring to the methane hydrate, is an ice-like clathrate constituted by hydrogen-bonded water molecules and light molecules like methane that have filled in the cavities via Van der Waals force (Sloan, 1998). This solidified natural gas (SNG) has been viewed as a potential alternative for natural gas transportation and storage because of several advantages (Thomas, 2003; Javanmardi et al., 2005; Koh et al., 2011; Veluswamy et al., 2018): the high volumetric storage capacity of 160–180 v/v, much milder formation and storing conditions than CNG and LNG, e.g., at 273.15 K and 3.2 MPa for methane hydrate formation, and safe and environmentally benign manufacturing process. However, technical challenges arise in the production process, primarily the slow kinetics of hydrate formation, large amount of heat generated, and limited gas storage capacity. Hydrate formation is always accompanied by heat release, which will impede hydrate growth if the heat is not removed in time, particularly in large-scale industrialization. Moreover, the theoretical gas storage capacity is hard to achieve due to the retarded mass transfer caused by the formation of thin hydrate layers at gas–liquid interfaces (Lee et al., 2006; Aman and Koh, 2016).

A great deal of effort has been focused on developing efficient methods for overcoming the above issues. To date, the most well-studied field is the formation of methane hydrates in the presence of surfactant, among which sodium dodecyl sulfate (SDS) showed the best performance (Zhong and Rogers, 2000; Kumar et al., 2015). In a recent review article, He et al. He et al. (2019) have provided a good review of surfactant-promoted gas hydrate formation during the past three decades. Given the enormous amount of foam production in hydrate dissociation and the difficulty of recycling the surfactant, non-surfactant-based methods for improving hydrate formation have attracted growing attention over the last 10 years. A review by Veluswamy et al. (2018) documented and discussed in detail the different materials applied for methane hydrate formation, e.g., silica gel, dry water, dry gel, sand, zeolite, and hollow silica, which are used as a fixed bed for hydrate reaction. Another review conducted by Nashed et al. (2018) shed light on the nanomaterials for gas hydrate formation, where various metal-based particles, like nano-Ag, Cu, CuO, and ZnO were discussed, and it was concluded that nanoparticles not only could help to promote mass transfer but they could also contribute to heat transfer enhancement in the hydrate reaction. Additionally, some non-metal materials such as silica nanoparticles (Wang et al., 2019), graphene (Wang et al., 2017), and carbon nanotubes (Pasieka et al., 2014) exhibited excellent performance in promoting gas storage capacities and hydrate formation rate.

As carbon-based materials (e.g., activated carbon, graphite, graphene, and carbon nanotubes) have been widely employed in gas hydrate formation in recent years, this mini-review summarizes the published studies where the promotion effects of carbon-based materials on gas hydrate formation were investigated. With an attempt to draw critical conclusions after compiling this knowledge into a single article, this review provides significant guidance for developing novel methods for hydrate-based technology.

## Gas Hydrate Formation With Carbon-Based Materials

Porous carbon-based materials, such as active carbon, graphite, carbon nanotubes, and graphene, can realize gas adsorption due to their porosity and high specific areas when utilized for hydrogen or methane storage (Nikitin et al., 2008; Mohan et al., 2019). During research on the gas adsorption process, scientists discovered that when carbon materials were wetted by water or dispersed in water, a higher methane storage capacity was obtained via hydrate formation under certain conditions. Hence, carbon materials attracted research interest as efficient promoters for the gas hydrate formation, resulting in numerous investigations in the last 10 years. Referring to the literature concerning different kinds of carbon materials, this section is divided into three parts: the promotion of gas hydrate formation by bulk carbon materials, carbon-based suspensions, and carbon-based nanofluid, respectively.

### Gas Hydrate Formation With Bulk Carbon Materials

Since natural gas hydrates are usually stored within porous sediments in nature, it is essential to understand the characteristics of hydrate formation in porous space. In experiments, the reactor is often filled with bulk materials with adsorbed water in the form of a packed bed for hydrate formation. The mass ratio of water to bulk materials, the material types, and the pore size are the primary factors that affect the gas hydrate formation rate and storage capacity.

The literature regarding the use of porous carbon materials (mainly referring to activated carbon) in methane hydrate formation is listed in chronological order in Table 1A. The porous material first reported as being for hydrate formation was active carbon, in an investigation by Zhou and Sun (2001). They found that wet activated carbon caused an increase in methane adsorption isotherms and enhanced gas uptake by 60% at a water ratio of 1.4. Later on, many studies proved the optimal water/carbon mass ratio to be about 1 (Perrin et al., 2003; Yan et al., 2005; Celzard and Marêché, 2006). By analysis of pore styles, Perrin et al. found that microporosity seemed to be useless for clathrate formation, while mesoporous and macroporous carbon materials were more favorable to enhancing hydrate formation. Following this work, Celzard and Marêché (2006) proved, however, that saturated pore space would slow down the hydrate formation kinetics since gas diffusion pathways became scarce when the small spaces in the pore network were filled by water. Similarly, another study showed that a 96.5% enhancement of water conversion was obtained due to the larger interstitial pore space between activated particles than between other smaller particles under 8 MPa and 4°C (Siangsai et al., 2015). Via observation of the morphology of methane hydrate formed in porous media of activated carbon, Babu et al. (2013) confirmed that the hydrates primarily nucleated on the surface of the activated carbon and that whether the hydrates further developed into stable hydrate crystals depended on the interstitial space between the activated carbon particles. As a consequence, porous activated carbons with an optimal water ratio can provide excellent interfaces that enlarge the area of gas–liquid contact for hydrate nucleation and growth, and the hydrate formation process is only accelerated by active carbons with large pore size rather than micropores.

**Table 1:** List of the carbon-based materials used in methane hydrate formation.

**Carbon material**	**Pore size (nm)**	**SSA (m^**2**^/g)**	**R_w_**	**T (K)**	**P (MPa)**	**References**
**(A)-FOR BULK CARBON-BASED MATERIALS**						
Activated carbon	-	1,800	1.4	275	4.6	Zhou and Sun, 2001
NC58	-	1,000	1.0	275.15	8	Perrin et al., 2003
NC86		1,257				
NC120		2,031				
Picazine		1,967				
Activated carbon	1.9	978, 1,126	1.7, 2.9	278	8	Yan et al., 2005
Activated carbon	-	1,000, 1,587, 2,031	1.09, 0.72, 0.85	275.15	8	Celzard and Marêché, 2006
NC120	-	2031	1.0	277.15	10	Najibi et al., 2008
Picazine		1967				
Activated carbon	2.19	866.7	0.5, 1.0	277.15	8	Babu et al., 2013
Activated carbon	-	864–918	1.0	277.15	6 or 8	Siangsai et al., 2015
Activated carbon	1.5	-	0.3	-	10	Liu et al., 2018
**Particles**	**Concentration**	**T (K)**	**P (MPa)**	**Duration (min)**	**Storage efficiency improvement**	**References**
**(B) FOR CARBON NANOTUBE-BASED NANOFLUID**						
OCNTs	0.001–0.006%	274.15	3&4	720	375%	Park et al., 2012
OCNTs	0.001–0.006%	274.15	-	720	260%	Pasieka et al., 2013
OCNTs	1 × 10^−6^–1 × 10^−4^%	275.15	4.7	-	-	Lim et al., 2014
SDS@ CNTs	0.05–0.6 mg/L	275.15	6	100	600%	Song Y. et al., 2017
RR195@CNTs	2–40 ppm	275.15	6	203	250%	Song et al., 2019
f-CNTs	10–150 ppm	275.15	6	100	575%	Song Y. M. et al., 2017
Ag@ OCNTs	20 ppm	275.15	6	110	650%	Song et al., 2018

*R_w_ is the mass ratio of water to carbon; “-” means “not found”; SSA refers to specific surface area; P and T are the pressure and temperature respectively; the storage efficiency improvement was calculated based on a pure water system*.

Aiming to determine the critical hydrate formation conditions, phase equilibrium estimations of gas hydrate formation in porous carbon materials have been conducted in many experimental or theoretical studies. The methane hydrate equilibrium was usually shifted to a higher pressure or lower temperature in bulk carbons compared to pure water (Najibi et al., 2008; Mingjun et al., 2010; Yang et al., 2012). For example, Liu et al. (2018) measured the methane hydrate formation or dissociation conditions in eleven porous materials, verifying that smaller pores size (below 6.2 nm) exerted a negative influence on the hydrate formation conditions due to extra capillary pressure in these pores. Taking the pore size, pore distribution, capillary pressure, and hydrate–liquid interfacial tension into consideration, some new equilibrium models were established and also supported the experimental results (Zhang et al., 2020).

The addition of a traditional promoter such as a surfactant or thermodynamic promoter into the water or offering hydrophobic/hydrophilic groups on the carbon surface have proved to be efficient ways of improving gas storage capacity and the hydrate formation rate in porous media (Casco et al., 2017; Cuadrado-Collados et al., 2020; Palodkar and Jana, 2020; Zhang et al., 2020). In the latest research, Cuadrado-Collados et al. (2020) reported the promotion effects of various additives, such as the sodium dodecyl sulfate (SDS), leucine, and tetrahydrofuran (THF) in the confined nanospace of the carbon surface, where hydrate nucleation and growth rate were both accelerated significantly. A similar work was conducted by Zhang et al. (2020), who noted that, when anionic active groups aggregated onto the surface of the porous media, the modified carbon could promote gas adsorption and enhance formation process because of micellar solubilization in the presence of SDS. By introducing oxygen-containing groups on the activated carbon, the carbons performed better after being wetted by water, as shown by the result that the methane hydrate yield was elevated to 51% for oxidation-treated carbons under the conditions of 3.3 MPa and 2°C. It was assumed that the locations of the oxygen groups on the surfaces of carbons acted as nucleation centers for water clustering, which benefited further hydrate growth (Casco et al., 2017). Herein, after functionalization or being attached to other promoters, porous carbon materials provided more efficient reaction media for hydrate formation.

There are two basic kinds of promotion mechanism for hydrate formation in wetted porous carbon materials. The generally accepted mechanism is the interface adsorption theory (Zhou and Sun, 2001; Mingjun et al., 2010; Cuadrado-Collados et al., 2018; Andres-Garcia et al., 2019). Unlike in the gas–free water system, there are many voids among and inside the carbon particles when water is absorbed in porous activated carbon, and these will provide efficient contact areas for gas and water. The hydrate formation process can then be described as: liquid water films gradually form at the surface of the carbon interface, followed by hydrate formation after gas adsorption at the water–carbon interface. This theory also points out that methane hydrates tend to form in wider pores and the intersectional spaces between particles. Another promotion mechanism is the capillary effect caused by the pores or interstitial space in the porous media. As the capillary force can enhance liquid phase migration in the pores, continuous gas–liquid contact is realized, and hydrate formation distributions are changed constantly. This promotion mechanism became more obvious when surfactant was added to the porous materials (Zhang et al., 2020). However, in this light, a minimum pore size of about 3 nm is required for methane hydrate formation considering the hydrate crystal size. Conversely, in some cases, the pore capillary force was assumed to reduce the activity of the pore-confined water that hindered hydrate formation (Liu et al., 2018).

### Gas Hydrate Formation With Carbon-Based Suspensions

Suspensions formed by carbon particles in an aqueous solution are considered another potential reaction medium for rapid hydrate formation (Takahata et al., 2010; Govindaraj et al., 2015; Yu et al., 2016, 2018). In case of severe sedimentation of hydrophobic particles in the reaction system, mechanical agitation is necessary during the hydrate formation. A carbon-based suspension is preferable to bulk materials in a fixed bed as the hydrate reaction system, since there are three distinct advantages when particles are dispersed in a liquid phase: the greater gas–liquid contact area of stirred suspensions, a more uniformly distributed hydrate crystallization process, and the feasibility of a continuous production process (Govindaraj et al., 2015).

By investigating methane hydrate formation kinetics in an activated carbon particle suspension at loadings of 0.5, 1.0, and 2.0 wt%, Govindaraj et al. elucidated that suspensions with a higher fraction of activated carbon particles had stronger promotion effects on hydrate formation kinetics (Govindaraj et al., 2015). Meanwhile, a prominent positive correlation was established between the activated carbon concentration and the hydrate gas storage capacity, where the gas storage capacity was increased by 60% in a 2.0 wt% suspension compared to a pure water system. Although in several studies, the graphite had marginal promotion effects on methane hydrates, mixtures of graphite and other promoters, e.g., a mixture of graphite and hematite or a mixture of graphite and surfactant could lead to rapid hydrate formation (Takahata et al., 2010; Yu et al., 2016). Carbon nanotubes, in particular, attracted most interest for the excellent thermal properties reported in some literature. By adding multi-walled or single-walled carbon nanotubes to pure water, the gas consumption and hydrate reaction rate during hydrate formation were dramatically improved (Park et al., 2010). A comparative study on the enhanced formation of methane hydrate by different types of CNTs indicated that a shorter nucleation stage and more rapid growth process were obtained when short nanotubes (CM-95) rather CM-100 were applied as additives as a result of the larger specific area of the shorter MWCNTs (Kim et al., 2011).

In summary, carbon particle suspensions have obvious promotion effects on gas hydrate formation. The primary reason for this is the enlarged gas–liquid contact area provided by suspended particles, which leads to a mass transfer enhancement. However, it is noted that hydrate formation must be carried out with the aid of stirring, and it thus requires extra energy consumption and the use of an agitation apparatus.

### Gas Hydrate Formation With Carbon-Based Nanofluid

Nanofluid is actually a stable dispersion formed by nanoparticles dispersed homogeneously in an aqueous phase. Nanofluid is considered an excellent hydrate reaction medium based on its superior mass transfer and heat transfer properties (Li et al., 2017; Nashed et al., 2018). The behaviors of nanoparticles in nanofluid that promote hydrate formation are as follows. Firstly, the nanoparticles move like microstirrers in the liquid through Brownian motion, resulting in constant updating of the gas–liquid interface. Secondly, the nanoparticles have high specific surface areas and can thus offer plenty of nucleation sites for hydrate formation. Lastly, the continuous movement of carbon nanoparticles helps to remove the heat generated during hydrate formation. Carbon nanomaterials such as carbon nanotubes and graphene are more beneficial to heat transfer due to their intrinsic high thermal conductivity.

Nanofluid constituted by water-soluble carbon nanotubes has been verified to be an excellent promoter for gas hydrate formation (as listed in Table 1B). When an oxidized CNT nanofluid was used as the reaction system, the gas consumption was up to 4.5 times higher than in water (Park et al., 2012; Li et al., 2017). The promotion efficiency of chemically or physically treated CNT nanofluid exceeded that of pristine CNTs. For instance, acid-treated CNTs, SDS-coated CNTs and plasma-functionalized CNTs could efficiently reduce induction time, increase gas consumption, and enhance growth rate (Park et al., 2010; Pasieka et al., 2013, 2015). The promotion efficiency of the CNT-based nanofluid, however, is affected by the particle fraction, the surface functional groups, and the treatment methods. The best concentration of OCNTs for promoting the growth of methane hydrate was 0.003% in Park et al. (2010). In view of the marked reactivity of the sulfonate groups contained in SDS, some researchers have coated the CNT surfaces with SDS, long-chain polymers containing SO_3_-, or Reactive Red 195 molecules and then dispersed the CNTs in water for use as the reaction system. Hydrates formed in these nanofluids all exhibited gas storage capacities that were elevated to 140–150 v/v, and the hydrate reactions finished within 100 min (Song Y. et al., 2017; Song Y. M. et al., 2017; Song et al., 2019). Moreover, with the aid of a high-speed ball milling process, the obtained functionalized CNTs (such as RR195@CNTs) had excellent recycling performance in the hydrate formation process (Song et al., 2019). Due to the thermal conductivity of metal nanoparticles (nano-Ag or nano-Cu), a prepared compound nanofluid containing OCNTs grafted by metal nanoparticles had a stronger promotion effect than the one-component nanofluid, with the exception that the metal nanoparticle-grafted OCNT nanofluid was not as stable as an OCNT nanofluid (Song et al., 2018).

Since graphene has smooth surfaces and is easy to functionalize by sulfonate groups or to load with metal nanoparticles, this two-dimensional carbon material is also introduced to hydrate formation reactions. Wang et al. (2017) grafted sulfonate groups successfully to graphene by covalent bonding and used it in methane hydrate formation. The results showed that the promotion efficiency of SGO (sulfated graphene) was better than that of GO (oxidized graphene). In another work, nano-Ag coated SGO was prepared for methane hydrate formation, and a shorter hydrate formation stage was achieved compared to SGO (He and Wang, 2018).

Considering that the fraction of carbon nanoparticles in the nanofluid is far smaller, the promotion efficiency of the equivalent carbon-based material in nanofluid is superior to the materials in suspension or a packed bed. Besides, the stable carbon-based nanofluid has excellent recycling performance in repeated hydrate formation, which thus contributes to more economical hydrate production.

## Conclusion and Prospects

**Figure 1:**
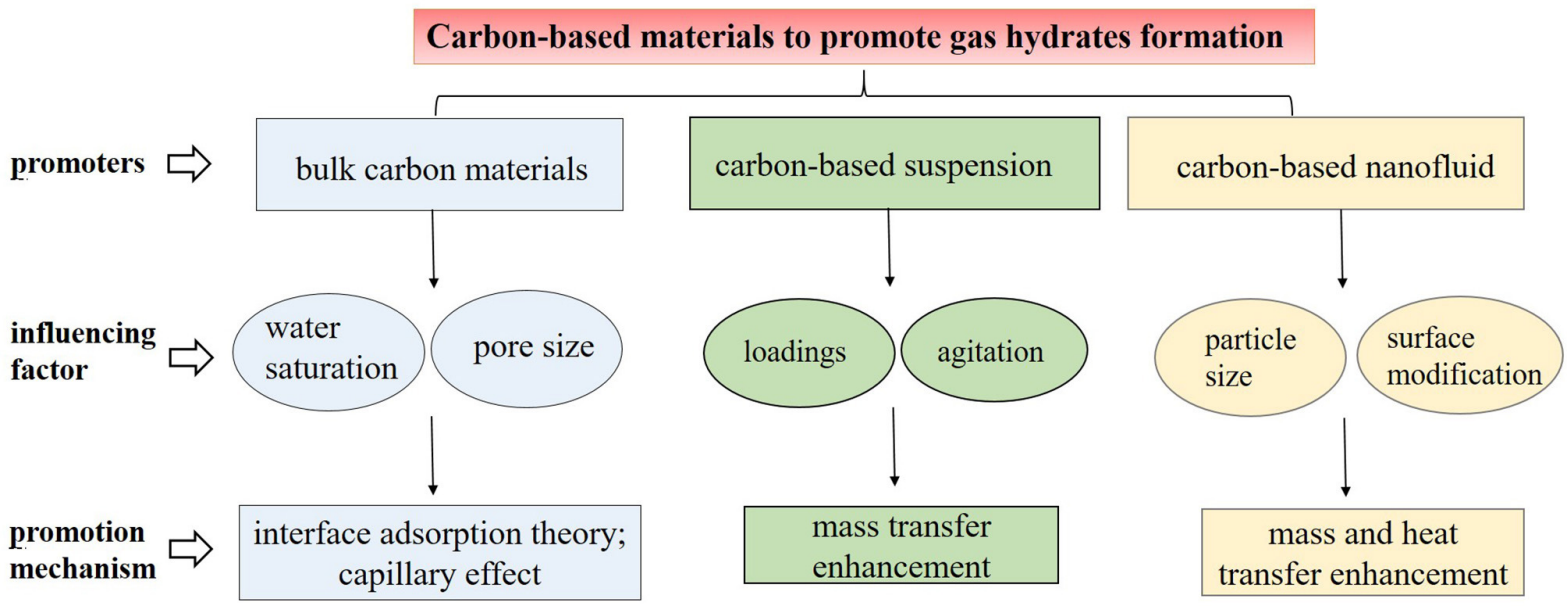
The schematic summary of the promotion of the carbon-based materials on the gas hydrate formation.

This work is devoted to the summary of hydrate formation in various carbon media of different forms: porous carbon materials in packed beds, particles in suspension, and nanoparticles in nanofluid. Figure 1 highlights the themes of this mini review. Porous carbons provide a large interface area for gas-liquid contact, and particles in suspension or nanofluids are helpful for heat and mass transfer enhancement. To sum up, carbon-based materials, either in macro or micro forms, all show unique promotion effects on gas hydrate formation. Carbon-based nanofluid is the preferable medium among these for achieving economical and efficient hydrate production. Accordingly, it is necessary to develop more economical and efficient carbon-based nanofluids via surface modifications or coupling with other promoters. Besides, a majority of current research focuses on experimental investigation, while few works have attempted molecular illustration of the gas hydrates promoted by those carbon materials. Molecular simulation or mathematical modeling to investigate the hydrate formation characteristics and hydrate growth mechanism in carbon-based materials is therefore required, and this would also be helpful for designing and propelling the application of novel carbon materials for hydrate-based technology.

## Author Contributions

Y-MS was in charge of literature collection, review, and writing. R-QL contributed to the tools and the internet search. FW and D-BZ helped write the manuscript. J-HS and LY assisted with manuscript enhancement. All authors contributed to the article and approved the submitted version.

## Conflict of Interest

The authors declare that the research was conducted in the absence of any commercial or financial relationships that could be construed as a potential conflict of interest.

## References

[B1] Aman Z. M. Koh C. A. (2016). Interfacial phenomena in gas hydrate systems. Chem. Soc. Rev. 45, 1678–1690. 10.1039/c5cs00791g 26781172

[B2] Andres-Garcia E. Dikhtiarenkob A. Fauthc F. Silvestre-Alberod J. Ramos-Fernándezd E. V. Jorge G. (2019). Methane hydrates Nucleation in microporous materials. Chem. Eng. J. 360, 569–576. 10.1016/j.cej.2018.11.216

[B3] Babu P. Yee D. Linga P. Palmer A. Khoo B. C. Tan T. S. (2013). Morphology of methane hydrate formation in porous media. Energ. Fuel. 27, 3364–3372. 10.1021/ef4004818

[B4] Casco M. E. Cuadrado-Collados C. Martínez-Escandell M. Rodríguez-Reinoso F. Silvestre-Albero J. (2017). Influences of the oxygen-containing surface functional groups in the methan hydrate nucleation and growth in nanoporous carbon. Carbon 123, 299–301. 10.1016/j.carbon.2017.07.061

[B5] Celzard A. Marêché J. F. (2006). Optimal wetting of active carbons for methane hydrate formation. Fuel 85, 957–966. 10.1016/j.fuel.2005.10.019

[B6] Cuadrado-Collados C. Farrando-Pérez J. Martínez-Escandell M. Missyul A. Silvestre-Albero J. (2020). Effect of additives in the nucleation and growth of methane hydrates confined in a high-surface area activated carbon material. Chem. Eng. J. 338, 124–224. 10.1016/j.cej.2020.124224

[B7] Cuadrado-Collados C. Fauth F. Such-Basanez I. Martínez-Escandell M. Silvestre-Albero J. (2018). Methane hydrate formation in the confined nanospace of activated carbons in seawater environment. Micropor. Mesopor. Mat. 255, 220–225. 10.1016/j.micromeso.2017.07.047

[B8] Govindaraj V. Mech D. Pandey G. Nagarajan R. Sangwai J. S. (2015). Kinetics of methane hydrate formation in the presence of activated carbon and nano-silica suspensions in pure water. J. Nat. Gas Sci. Eng. 26, 810–818. 10.1016/j.jngse.2015.07.011

[B9] He Y. Sun M.-T. Chen C. Zhang G.-D. Chao K. Lin Y. (2019). Surfactant-based promotion to gas hydrate formation for energy storage. J. Mater. Chem. A 7, 21634–21661. 10.1039/c9ta07071k

[B10] He Y. Wang F. (2018). Hydrate-based CO_2_ capture: kinetic improvement via graphene-carried –SO3- and Ag nanoparticles. J. Mater. Chem. A 6, 22619–22625. 10.1039/C8TA08785G

[B11] Javanmardi J. Nasrifar K. Najibi S. H. Moshfeghian M. (2005). Economic evaluation of natural gas hydrate as an alternative for natural gas transportation. Appl. Therm. Eng. 25, 1708–1723. 10.1016/j.applthermaleng.2004.10.009

[B12] Kim N. J. Park S. S. Kim H. T. Chun W. (2011). A comparative study on the enhanced formation of methane hydrate using CM-95 and CM-100 MWCNTs. Int. Commun. Heat Mass 38, 31–36. 10.1016/j.icheatmasstransfer.2010.10.002

[B13] Koh C. A. Sloan E. D. Sum A. K. Wu D. T. (2011). Fundamentals and Applications of Gas Hydrates. Annu. Rev. Chem. Biomol. 22, 237–257. 10.1146/annurev-chembioeng-061010-114152 22432618

[B14] Kumar A. Bhattacharjee G. Kulkarni B. D. Kumar R. (2015). Role of Surfactants in Promoting Gas Hydrate Formation. Ind. Eng. Chem. Res. 54, 12217–12232. 10.1021/acs.iecr.5b03476

[B15] Lee J. D. Song M. Susilo R. Englezos P. (2006). Dynamics of methane–propane clathrate hydrate crystal growth from liquid water with or without the presence of n-heptane. gryst. Growth Des. 6, 1428–1439. 10.1021/cg0600647

[B16] Li D. L. Peng H. Liang D. Q. (2017). Thermal conductivity enhancement of clathrate hydrate with nanoparticles. Int. J. Heat Mass Tran. 104, 566–573. 10.1016/j.ijheatmasstransfer.2016.08.081

[B17] Lim S. H. Riffat S. B. Park S. S. Oh S.-J. Chun W. Kim N.-J. (2014). Enhancement of methane hydrate formation using a mixture of tetrahydrofuran and oxidized multi-wall carbon nanotubes. Int. J. Energ. Res. 38, 374–379. 10.1002/er.3051

[B18] Liu H. Zhan S. Guo P. Fan S. Zhang S. (2018). Understanding the characteristic of methane hydrate equilibrium in materials and its potential application. Chem. Eng. J. 349, 775–781. 10.1016/j.cej.2018.05.150.2018.05.150

[B19] Mingjun Y. Yongchen S. Liu Y. Yongjun C. Qingping L. (2010). Influence of pore size, salinity and gas composition upon the hydrate formation conditions. Therm. Chem. Eng. Data 18, 292–296. 10.1016/S1004-9541(08)60355-9

[B20] Mohan M. Sharma V. K. Kumar E. A. Gayathri V. (2019). Hydrogen storage in carbon materials—A review. Energy Storage 1:35 10.1002/est2.35

[B21] Najibi H. Chapoy A. Tohidi B. (2008). Methane_natural gas storage and delivered capacity for activated carbons in dry and wet conditions. Fuel 87, 7–13. 10.1016/j.fuel.2007.03.044

[B22] Nashed O. Partoon B. Lal B. Sabil K. M. Shariff A. M. (2018). Review the impact of nanoparticles on the thermodynamics and kinetics of gas hydrate formation. J. Nat. Gas Sci. Eng. 55, 452–465. 10.1016/j.jngse.2018.05.022

[B23] Nikitin A. Li X. Zhang Z. Ogasawara H. Dai H. Nilsson A. (2008). Hydrogen storage in carbon nanotubes through the formation of stable C-H bonds. Nano Lett. 8, 162–167. 10.1021/nl072325k 18088150

[B24] Palodkar A. V. Jana A. K. (2020). Clathrate hydrate dynamics with synthetic and bio-surfactant in porous media: model formulation and validation. Chem. Eng. Sci. 213:115386 10.1016/j.ces.2019.115386

[B25] Park S. S. An E. J. Lee S. B. Chun W. G. Kim N. J. (2012). Characteristics of methane hydrate formation in carbon nanofluids. J. Ind. Eng. Chem. 18, 443–448. 10.1016/j.jiec.2011.11.045

[B26] Park S. S. Lee S. B. Kim N. J. (2010). Effect of multi-walled carbon nanotubes on methane hydrate formation. J. Ind. Eng. Chem. 16, 551–555. 10.1016/j.jiec.2010.03.023

[B27] Pasieka J. Coulombe S. Servio P. (2013). Investigating the effects of hydrophobic and hydrophilic multi-wall carbon nanotubes on methane hydrate growth kinetics. Chem. Eng. Sci. 104, 998–1002. 10.1016/j.ces.2013.10.037

[B28] Pasieka J. Coulombe S. Servio P. (2014). The effect of hydrophilic and hydrophobic multi-wall carbon nanotubes on methane dissolution rates in water at three phase equilibrium (V-L-w-H) conditions. Ind. Eng. Chem. Res. 53, 14519–14525. 10.1021/ie502457c

[B29] Pasieka J. Jorge L. Coulombe S. Servio P. (2015). Effects of as-produced and amine-functionalized multi-wall carbon nanotubes on carbon dioxide hydrate formation. Energ. Fuel. 29, 5259–5266. 10.1021/acs.energyfuels.5b01036

[B30] Perrin A. Celzard A. Marêch,é J. F. Furdin G. (2003). Methane storage within dry and wet active carbons a comparative study. Energ. Fuel. 17, 1283–1291. 10.1021/ef030067i

[B31] Siangsai A. Rangsunvigit P. Kitiyanan B. Kulprathipanja S. Linga P. (2015). Investigation on the roles of activated carbonparticle sizes on methane hydrate formation and dissociation. Chem. Eng. Sci. 126, 383–389. 10.1016/j.ces.2014.12.047

[B32] Sloan E. D. (1998). Clathrate Hydrate of Natural Gases. New York, NY: Marcel Dekker.

[B33] Song Y. Wang F. Liu G. Q. Luo S. J. Guo R.B. (2017). Promotion efffect of nanotubes-doped SDS on the methane hydrate formation. Energ.Fuel. 31, 1850–1857. 10.1021/acs.energyfuels.6b02418

[B34] Song Y. M. Wang F. Guo G. Luo S. J. Guo R. B. (2017). Amphiphilic-polymer-coated carbon nanotubes as promoters for methane hydrate formation. ACS Sustain Chem Eng. 5, 9271–9278. 10.1021/acssuschemeng.7b02239

[B35] Song Y. M. Wang F. Luo S. J. Guo R. B. Xu D. (2019). Methane hydrate formation improved by water-soluble carbon nanotubes via π-π conjugated molecules functionalization. Fuel 243, 185–191. 10.1016/j.fuel.2019.01.081

[B36] Song Y. M. Wang F Guo G. Luo S.-J. Guo R.-B. (2018). Energy-efficient storage of methane in the formed hydrates with metal nanoparticles-grafted carbon nanotubes as promoter. Appl. Energ. 224, 175–183. 10.1016/j.apenergy.2018.04.068

[B37] Takahata M. Kashiwaya Y. Ishi K. (2010). Kinetics of methane hydrate formation catalyzed by iron oxide and carbon under intense stirring conditions. Mater Trans 51, 727–734. 10.2320/matertrans.M2009369

[B38] Thomas S. (2003). Review of ways to transport natural gas energy from countries which do not need the gas for domestic use. Energy 28, 1461–1477. 10.1016/s0360-5442(03)00124-5

[B39] Veluswamy H. P. Kumar A. Seo Y. Lee J. D. Linga P. (2018). A review of solidified natural gas (SNG) technology for gas storage via clathrate hydrates. Appl. Energ. 216, 262–285. 10.1016/j.apenergy.2018.02.059

[B40] Wang F. Meng H. L. Guo G. Luo S. J. Guo R. B. (2017). Methane hydrate formation promoted by -SO3–coated graphene oxide nanosheets. ACS sustain. Chem. Eng. 5, 6597–6604. 10.1021/acssuschemeng.7b00846

[B41] Wang R. Liu T. Ning F. Ou W. Zhang L. Wang Z. (2019). Effect of hydrophilic silica nanoparticles on hydrate formation: insight from the experimental study. J. Energ Chem. 30, 90–100. 10.1016/j.jechem.2018.02.021

[B42] Yan L. Chen G. Pang W. Liu J. (2005). Experimental and modeling study on hydrate formation in wet activated carbon. J. Phys. Chem. B 109, 6025–6030. 10.1021/jp045679y 16851658

[B43] Yang M. Song Y. Ruan X. Liu Y. Zhao J. Li Q. (2012). Thermodynamic modeling of pure components including the effects of capillarity. Energies 5, 925–937. 10.3390/en5040925

[B44] Yu Y. S. Xu C. G. Li X. S. (2018). Evaluation of CO_2_ hydrate formation from mixture of graphite nanoparticle and sodium dodecyl benzene sulfonate. J. Ind. Eng. Chem. 59, 64–69. 10.1016/j.jiec.2017.10.007

[B45] Yu Y. S. Zhou S. D. Li X. S. Wang S. L. (2016). Effect of graphite nanoparticles on CO_2_ hydrate phase equilibrium. Fluid Phase Equilibr. 414, 23–28. 10.1016/j.fluid.2015.12.054

[B46] Zhang Z. Liu Z. Pan Z. Baena-Morenoe F. M. Soltanian M. R. (2020). Effect of porous media and its distribution on methane hydrate formation in the presence of surfactant. Appl. Energ. 261:114373 10.1016/j.apenergy.2019.114373

[B47] Zhong Y. Rogers R. E. (2000). Surfactant effects on gas hydrate formation. Chem. Eng. Sci. 55, 4175–4187. 10.1016/S0009-2509(00)00072-5

[B48] Zhou L. Sun Y. (2001). Enhancement of the methane storage on activated carbon by preadsorbed water. AIChE J. 48, 2412–2416. 10.1002/aic.690481030

